# The association between maternal use of folic acid supplements during pregnancy and risk of autism spectrum disorders in children: a meta-analysis

**DOI:** 10.1186/s13229-017-0170-8

**Published:** 2017-10-02

**Authors:** Meiyun Wang, Kaiqin Li, Dongmei Zhao, Ling Li

**Affiliations:** 1 Department of Pediatric Health Care, Ji’nan Children’s Hospital of Shandong University, No. 23976, Jingshi Road, Ji’nan, Shandong Province 250022 China; 2 Department of Gynecology, The Central Hospital of Linyi City, Linyi, Shandong 276000 China

**Keywords:** Maternal, Folic acid supplements, Autism spectrum disorders, Children, Meta-analysis

## Abstract

Previous reviews have been conducted to evaluate the association between maternal use of folic acid supplements during pregnancy and risk of autism spectrum disorders (ASD) in children, with no definitive conclusion. We therefore conducted a more comprehensive meta-analysis to reassess the relationship between folic acid and the risk of ASD. The electronic databases PubMed, Web of Knowledge, and Wanfang Data were carefully searched to find eligible studies as recent as March 2017. A random effects model was used to combine the relative risk (RR) with 95% confidence intervals (CI). Sensitivity analysis and publication bias were conducted. A total of 12 articles with 16 studies comprising 4514 ASD cases were included in this report. It was found that supplementation with folic acid during pregnancy could reduce the risk of ASD [RR = 0.771, 95% CI = 0.641–0.928, *I* ^*2*^ = 59.7%, *P*_heterogeneity_ = 0.001] as compared to those women without folic acid supplementation. The associations were significant among Asian, European, and American populations. In summary, this comprehensive meta-analysis suggested that maternal use of folic acid supplements during pregnancy could significantly reduce the risk of ASD in children regardless of ethnicity, as compared to those women who did not supplement with folic acid.

**Electronic supplementary material:**

The online version of this article (10.1186/s13229-017-0170-8) contains supplementary material, which is available to authorized users.

We began our exploration into this topic through a study entitled “Environmental risk factors for autism: an evidence-based review of systematic reviews and meta-analyses” [1]. Within that paper, the authors analyzed the environmental risk factors for autism, including maternal use of folic acid supplements during pregnancy. Additionally, a study had been conducted to assess the association between folic acid and autism, which investigated that specific relationship more thoroughly [2]. However, these two previous studies did not combine their results using a meta-analytic approach. Therefore, the aim of this article is to establish a more definitive conclusion on the association between maternal use of folic acid supplements during pregnancy and autism spectrum disorder (ASD) risk in children.

The databases of PubMed, Web of Knowledge, and Wanfang Database were searched extensively to find eligible studies as recent as March 2017. The following search terms were used: [“folate” OR “folic acid” OR “vitamins” OR “diet”] AND [“Autism” OR “Autism spectrum disorder” OR “ASD”]. Articles were included if they presented data regarding the association between folic acid supplements during pregnancy and risk for ASD. Studies were excluded if they were written in languages other than English and Chinese, did not offer enough data, were reviews, editorials, comments, or if the studies were not conducted with humans as the test subjects. Two independent investigators searched and reviewed all identified studies, then extracted the data reported in Additional file 1: Table S1.

The DerSimonian and Laird random effects model was adopted to pool the study-specific relative risk (RR) with their 95% confidence intervals (CI) on the association between maternal use of folic acid supplements during pregnancy and ASD risk, which considers both within-study and between-study variation [3]. Heterogeneity across studies was checked by the chi-square test and *I* ^2^ test, and *I* ^2^ values of 0, 25, 50, and 75% represent no, low, moderate, and high heterogeneity, respectively [4, 5]. Sensitivity analysis was performed to assess whether the results could be affected markedly when one study was removed at a time [6]. Small study effect was assessed with visual inspection of Egger’s test and funnel plot [7]. Data was analyzed using STATA version 12.0 (Stata Corporation, College Station, TX, USA). *P* ≤ 0.05 (two-tailed) was accepted as statistically significant for computed effects.

The flowchart of this meta-analysis for exclusion/inclusion of studies is shown in Additional file 2: Figure S1. A total of 12 articles [8–19] with 16 studies comprising 4514 ASD cases were included in this report. Overall, maternal use of folic acid supplements during pregnancy could reduce the risk of ASD [RR = 0.771, 95% CI = 0.641–0.928, *I* ^*2*^ = 59.7%, *P*_heterogeneity_ = 0.001, Fig. 1] as compared to those with no folic acid supplementation. Inverse relationships were found both in prospective studies [RR = 0.903, 95% CI = 0.786–0.998] and in case-control studies [RR = 0.435, 95% CI = 0.264–0.717]. The associations were also significant among Asian populations [RR = 0.669, 95% CI = 0.461–0.972], European populations [RR = 0.844, 95% CI = 0.683–0.993], and American populations [RR = 0.405, 95% CI = 0.165–0.994] (Fig. 1). The detailed subgroup analyses are showed in Table 1.

**Fig. 1:**
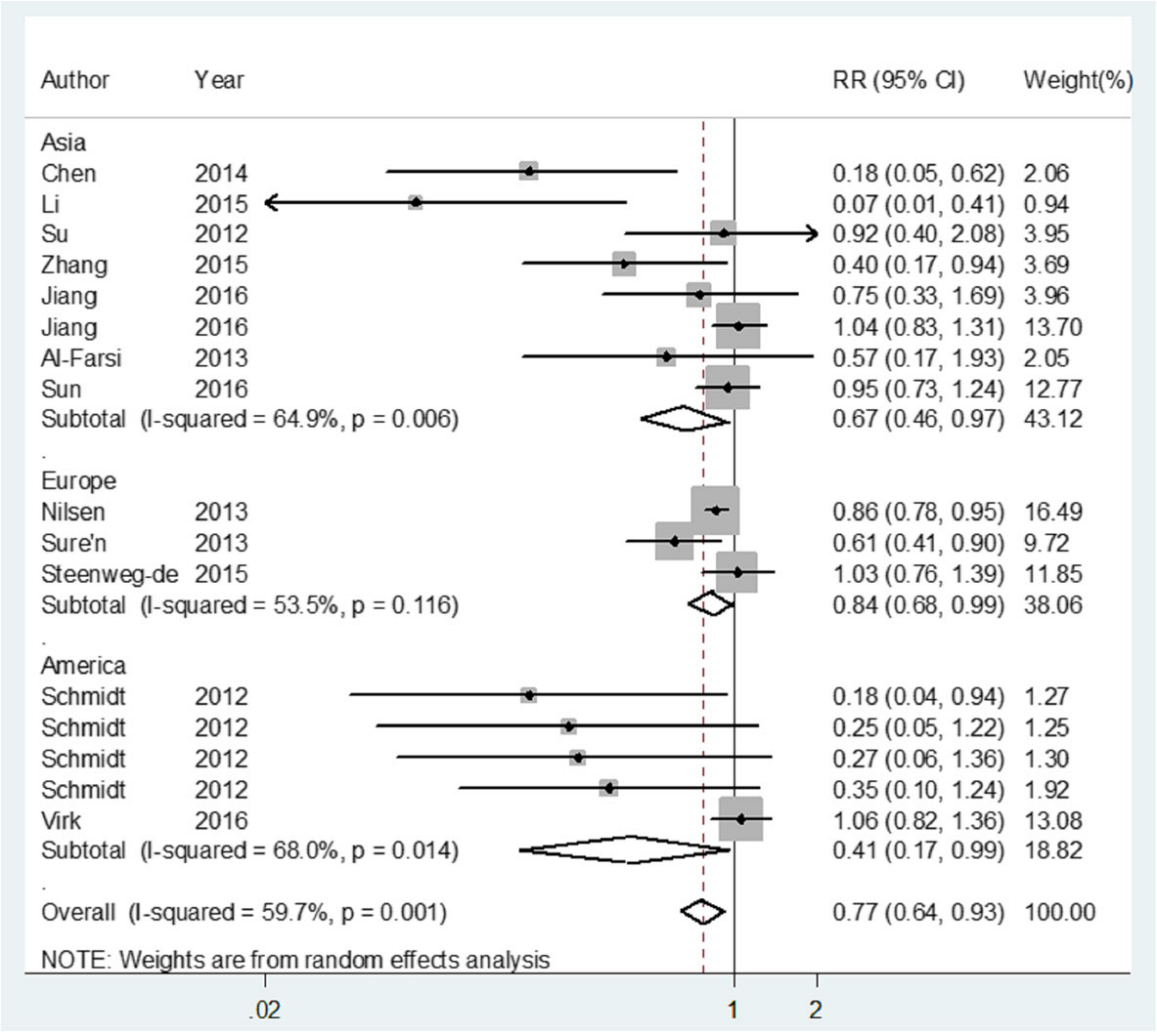
The forest plot of the association between maternal use of folic acid supplements during pregnancy compared with those no supplements, subgroup by ethnicity

**Table 1:** Summary risk estimates of the overall and subgroup analyses on maternal use of folic acid supplements during pregnancy and risk of autism spectrum disorders in children

Subgroups	No. (cases)	No. (studies)	Risk estimate (95% CI)	Heterogeneity test *I* ^2^ (%) *P* value	
All studies	4514	16	0.771(0.641–0.928)	59.7	0.001
Study design					
Prospective	2794	5	0.903(0.786–0.998)	42.6	0.138
Case-control	1720	11	0.435(0.264–0.717)	66.0	0.001
Ethnicity					
Asian	1581	8	0.669(0.461–0.972)	64.9	0.006
European	2258	3	0.844(0.683–0.993)	53.5	0.116
American	675	5	0.405(0.165–0.994)	68.0	0.014

In the pooled result for all of the studies included, evidence of between-study heterogeneity was demonstrated. We therefore used univariate meta-regression to explore the reason for this high heterogeneity with the covariates of publication year, number of cases, study type, the time of folic acid supplement intake, the method of folic acid supplementation, and geographic locations. No significant finding was found contributing significantly to the between-study heterogeneity. Begg’s funnel plot (Additional file 3: Figure S2) and Egger’s test (*P* = 0.112) indicated that there was no significant publication bias. Sensitivity analysis showed that no single study had excessive influence on the pooled result while excluding one study at a time (Additional file 4: Figure S3).

One limitation of our report is that mothers were asked to recall a period several years before the interview when reporting on folic acid and supplement information. Given the elapsed time of the period of recall, inaccuracies would be expected. Another limitation is the significant heterogeneity observed in the result. The between-study heterogeneity was not resolved by meta-regression, and the results of subgroup analyses also demonstrated high heterogeneity. Therefore, other genetic and environment variables, as well as their possible interactions, may be potential contributors to the heterogeneity found.

In summary, this comprehensive meta-analysis suggested that maternal use of folic acid supplements during pregnancy could significantly reduce the risk of ASD in children as compared to those without folic acid supplementation. Significant associations were found in Asian, European, and American populations. Since some limitations are present in our study, future studies should aim to investigate the effect of the timing of folic acid supplement intake during the pregnancy, as well as the effects of varying dosages.

## Additional files

Additional file 1: Table S1. Characteristics of the included studies on maternal use of folic acid supplements during pregnancy and risk of autism spectrum disorders in children. (DOC 49 kb)

Additional file 2: Figure S1. Flowchart of study selection. (TIFF 102 kb)

Additional file 3: Figure S2. Begg’s funnel plot of the association between maternal use of folic acid supplements during pregnancy compared with those no supplements. (TIFF 116 kb)

Additional file 4: Figure S3. Sensitivity analysis of the association between maternal use of folic acid supplements during pregnancy compared with those no supplements. (TIFF 8 kb)

## Abbreviations

ASD: Autism spectrum disorders
CI: Confidence intervals
RR: Relative risk
