# Supplementary material for: The association between maternal use of folic acid supplements during pregnancy and risk of autism spectrum disorders in children: a meta-analysis
Source: Mol Autism. 2017 Oct 2;8:51. doi: 10.1186/s13229-017-0170-8 (PMC5625821; doi:10.1186/s13229-017-0170-8)
Supplement: Supplementary file 1 — Characteristics of the included studies on maternal use of folic acid supplements during pregnancy and risk of autism spectrum disorders in children. (DOC 49 kb) [file 13229_2017_170_MOESM1_ESM.doc]

**Table S1** Characteristics of the included studies on maternal use of folic acid supplements during pregnancy and risk of autism spectrum disorders in Children

| Study,  year | Country | Female  rate(%) | Way of diagnosed | Age | Study design  (follow-up duration) | Participants  (cases) | Folic acid supplementation time | RR (95%CI) for folic acid supplements compared with no supplements | Ways of folic  acid supplement | Control  sample |
| --- | --- | --- | --- | --- | --- | --- | --- | --- | --- | --- |
| Chen et al.  2014 | China | 14.29 | DSM-IV | 1.5~3 | Case-control | 4740  (14) | During pregnancy | 0.18 (0.06-0.62) | Folic acid specific vitamins | Children without ASD by DSM diagnosis |
| Li et al.  2015 | China | 12.71 | DSM-IV | 1~5 | Case-control | 362  (181) | Before pregnancy and early pregnancy | 0.07 (0.01-0.41) | Folic acid specific vitamins | Health children |
| Su et al.  2012 | China | 29.41 | CARS and  DSM-IV | 13.40  ±4.27 | Case-control | 297  (96) | During pregnancy | 0.92 (0.40-2.08) | Folic acid specific vitamins | Health children |
| Zhang et al.  2015 | China | 19.23 | CARS and  DSM-IV | 8.90  ±4.39 | Case-control | 733  (193) | During pregnancy | 0.40 (0.17-0.94) | Folic acid specific vitamins | Health children |
| Jiang et al.  2016 | China | 45.60 | ABC | 2.5~3.5 | Case-control | 8075  (767) | During pregnancy | Subclinical group:  0.75 (0.33-1.69)  Suspected clinical group: 1.04 (0.83-1.31) | Folic acid specific vitamins | Children without ASD |
| Nilsen et al.  2013 | Norway | 48.70 | ABC and  DSM-IV | 3~11 | Cohort  (3) | 507856  (2072) | During pregnancy | Medical birth registry of Norway cohort:  0.86 (0.78-0.95) | Folic acid specific vitamins | Children without ASD |
| Sure’n et al.  2013 | Norway | NA | DSM-IV | 3.3~10.2 | Cohort  (4) | 85176  (114) | 4 weeks before to 8 weeks after pregnancy | 0.61 (0.41-0.9) | Folic acid specific vitamins | Children without ASD |
| Schmidt et  al. 2012 | USA | 13.30 | ADI-R and ADOS | 2~5 | Case-control | 707  (429) | 3 months before to all pregnancy | ＞1000 ug/d:  0.18 (0.04-0.94)  800-1000 ug/d:  0.25 (0.05-1.22)  500-800ug/d:  0.27 (0.06-1.36)  ≤500 ug/d:  0.35 (0.10-1.24) | Dietary folic acid supplementation | Children without ASD diagnosis by ADI-R and ADOS |
| Virk et al.  2016 | USA | 48.80 | ICD-10 | 8.1~11.4 | Cohort  (9.6) | 15246  (246) | 4 weeks before to 8 weeks after pregnancy | 1.06 (0.82-1.36) | Folic acid specific vitamins | Children without ASD |
| Steenweg-de  et al. 2015 | Netherland | 49.80 | DSM-IV and SRS | 6.2 ± 0.5 | Cohort  (NA) | 3893  (72) | 4 weeks before to 8 weeks after pregnancy | 1.03 (0.76-1.39) | Folic acid specific vitamins | Children without ASD |
| Al-Farsi et  al. 2013 | Oman | 50.00 | DSM-IV | 3~5 | Case-control | 80  (40) | During pregnancy | 0.57 (0.17-1.93) | Dietary folic acid supplementation | Health children |
| Sun et al.  2016 | China | 46.30 | CABS | 3~6 | Cohort  (3.5) | 4740  (290) | Early pregnancy | 0.95 (0.73-1.24) | Folic acid specific vitamins | Children without ASD |

Abbreviations: CI=confidence interval; RR=relative risk; DSM-IV = Diagnostic and Statistical Manual of Mental Disorders (Fourth Edition); CARS= Childhood Autism Rating Scale; ASD= autism spectrum disorders; ABC= Autism Behavior Checklist; ADI-R= Autism Diagnostic Interview–Revised; ADOS= Autism Diagnostic Observation Schedule–Generic; ICD-10= International Classification of Diseases-10; SRS= Social Responsiveness Scale; CABS= Clancy Autism Behavior Scale; NA= not available.
