# Supplementary figures and images for: The association between maternal use of folic acid supplements during pregnancy and risk of autism spectrum disorders in children: a meta-analysis
Source: Mol Autism. 2017 Oct 2;8:51. doi: 10.1186/s13229-017-0170-8 (PMC5625821; doi:10.1186/s13229-017-0170-8)

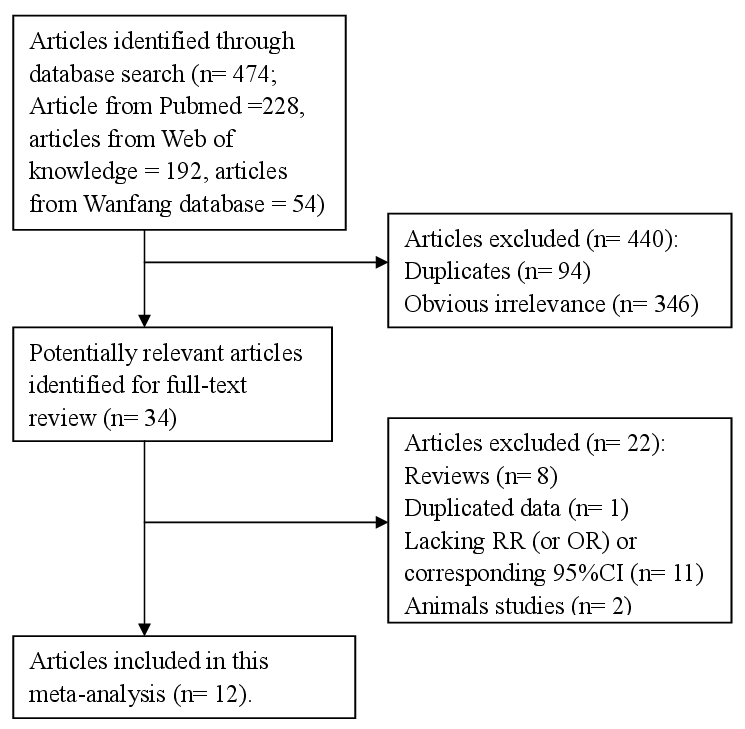

Supplement: Supplementary file 2 — Flowchart of study selection. (TIFF 102 kb) [file 13229_2017_170_MOESM2_ESM.tif]

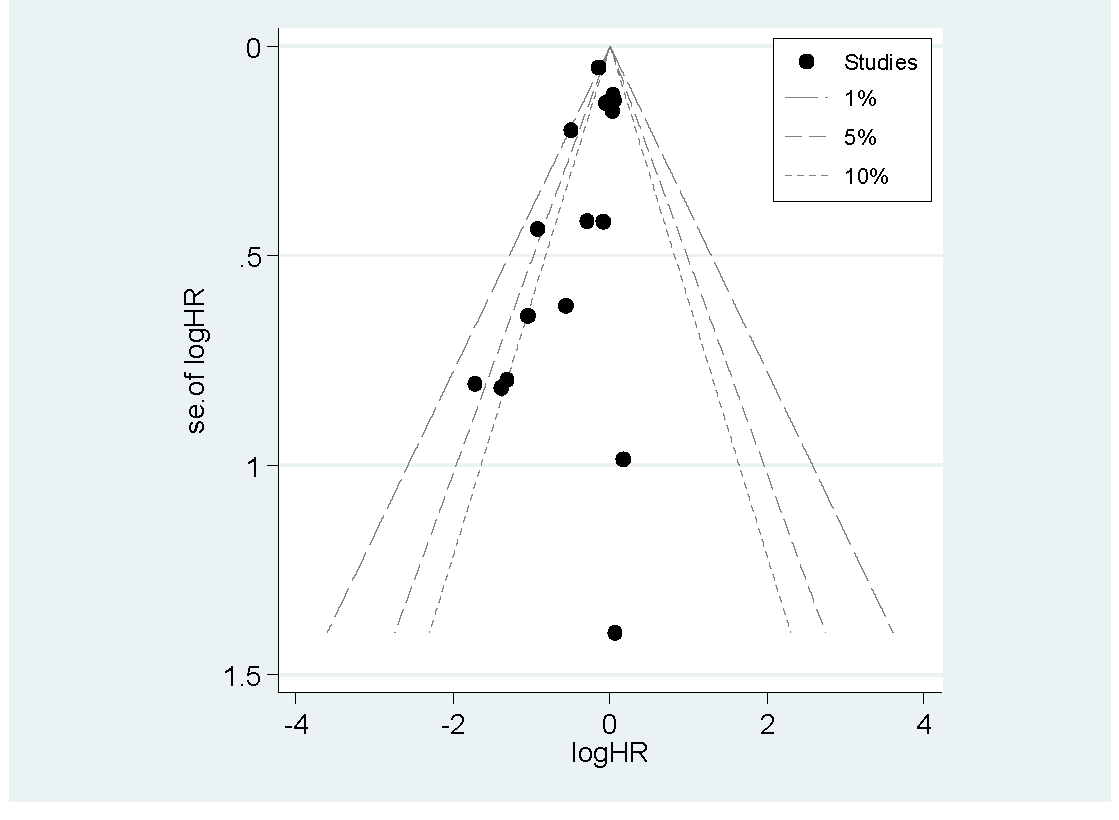

Supplement: Supplementary file 3 — Begg’s funnel plot of the association between maternal use of folic acid supplements during pregnancy compared with those no supplements. (TIFF 116 kb) [file 13229_2017_170_MOESM3_ESM.tif]
